# Synthesis and Transport Properties of Novel MOF/PIM-1/MOF Sandwich Membranes for Gas Separation

**DOI:** 10.3390/membranes7010007

**Published:** 2017-02-11

**Authors:** Alessio Fuoco, Muhanned R. Khdhayyer, Martin P. Attfield, Elisa Esposito, Johannes C. Jansen, Peter M. Budd

**Affiliations:** 1 Institute on Membrane Technology, ITM-CNR, Via P. Bucci 17/C, Rende (CS) 87036, Italy; a.fuoco@itm.cnr.it (A.F.); e.esposito@itm.cnr.it (E.E.); 2 School of Chemistry, University of Manchester, Manchester M13 9PL, UK; mradhi.2008@gmail.com (M.R.K.); m.attfield@manchester.ac.uk (M.P.A.)

**Keywords:** composite membrane, polymer of intrinsic microporosity, metal-organic framework, gas permeation

## Abstract

Metal-organic frameworks (MOFs) were supported on polymer membrane substrates for the fabrication of composite polymer membranes based on unmodified and modified polymer of intrinsic microporosity (PIM-1). Layers of two different MOFs, zeolitic imidazolate framework-8 (ZIF-8) and Copper benzene tricarboxylate ((HKUST-1), were grown onto neat PIM-1, amide surface-modified PIM-1 and hexamethylenediamine (HMDA) -modified PIM-1. The surface-grown crystalline MOFs were characterized by a combination of several techniques, including powder X-ray diffraction, infrared spectroscopy and scanning electron microscopy to investigate the film morphology on the neat and modified PIM-1 membranes. The pure gas permeabilities of He, H_2_, O_2_, N_2_, CH_4_, CO_2_ were studied to understand the effect of the surface modification on the basic transport properties and evaluate the potential use of these membranes for industrially relevant gas separations. The pure gas transport was discussed in terms of permeability and selectivity, highlighting the effect of the MOF growth on the diffusion coefficients of the gas in the new composite polymer membranes. The results confirm that the growth of MOFs on polymer membranes can enhance the selectivity of the appropriately functionalized PIM-1, without a dramatic decrease of the permeability.

## 1. Introduction

Polymeric membranes for gas separation are used for highly energy-efficient industrial separations. Currently, they are employed in a broad range of applications, such as O_2_ enrichment of air and purification of natural and biogas, and they show potential for many other commercially interesting applications, such as CO_2_ removal/capture from pre and post combustion streams. Both industrial and academic research laboratories are focusing their interest in the design of new membranes that could surpass the empirical Robeson’s trade-off between permeability and selectivity [1,2]. In the last decade, several materials of potential interest for membrane preparation have been developed, such as polymers of intrinsic microporosity (PIMs) and metal-organic frameworks (MOFs). The latter have been incorporated as fillers in many different polymer matrices for fabrication of hybrid composite membranes or mixed matrix membranes (MMMs), to enhance their permeability and selectivity. A reason to incorporate highly permeable MOFs in the polymer matrix is also to limit the impact of physical aging [3], which may significantly reduce the permeability as a function of time in PIMs [4]. However, it is challenging to form a uniform dispersion of the MOF in these MMMs [5]. This is often due to the poor compatibility between the MOF particles and the polymer matrix, leading to agglomeration of the MOF, particularly at higher filler loadings, or to sedimentation, at low polymer concentration and low viscosity of the casting solution. This results in poor membrane performance due to the formation of non-selective interfacial voids [6]. The latter is an important potential drawback of MMMs, because it is difficult to increase the MOF loading. High filler loadings in the polymer matrix can establish percolative network pathways to enhance gas separation performance, provided non-selective voids resulting in a loss of selectivity can be avoided [7,8]. However, high filler loadings can lead to brittle MMMs with poor mechanical properties. Besides these issues, the pores of MOF fillers can be blocked by polymer chains, when mixed together, which hinders the direct contact of gas molecules with the internal void space of the MOF particles [8,9]. In the last few years, various MMMs have been reported, based on PIM-1 and different inorganic [10], carbonaceous [11,12], organic [3] or MOF fillers [13,14,15] or a MOF/ionic liquid combination [16], with different effects on the gas permeability and selectivity.

This paper proposes a new approach to develop symmetric composite MOF based membranes by growing MOF particles on the surface of polymer membranes as an alternative to their dispersion inside the polymer matrix [17]. Surface modification of the polymer membrane can enhance the compatibility by interaction of the metal ions and the organic ligands of the MOF with the functional groups on the surface of the polymer membrane. In addition, the MOF loading can be increased by controlling the concentration of the MOF precursors or increasing the number of growth cycles [5,17]. The new class of MOF/polymer/MOF sandwich membranes should be less brittle compared to MOF based MMMs, because the polymer support retains most of its flexibility and the polymer/MOF sandwich membranes can be reasonably bent or tailored to some extent [8,18].

Many different synthetic techniques have been reported to deposit a MOF film on solid surfaces, including surface functionalization of the solid substrates to form self-assembled monolayers (SAMs) [5,19,20,21,22,23,24,25]. These SAMs provide active sites for promoting the heterogeneous nucleation and the growth of MOF crystals and enhance the adhesion between MOF particles and supports as well [17,26]. In general, fabrication of thin films of crystalline MOF follows one of two approaches that are the most commonly used methods: (1) Layer-by-Layer (LbL) growth or Step by Step deposition of crystals on the surface of substrate; (2) In situ growth (ISG) or direct growth/deposition from MOF growth precursor solutions.

The LbL growth method is carried out by dipping the functionalized substrate sequentially into the two separate MOF precursor solutions, the metal ion solution and the organic ligand solution. Between each step the substrate is washed with solvent to remove the uncoordinated metal ions or organic linkers [27,28]. This method separates the nucleation step and the growth step in the MOF fabrication. It has unique advantages that a wide variety of both organic and inorganic substrates can be coated by LbL thin films and that the film thickness is largely controlled by the number of deposition cycles. Compared with other methods for thin film fabrication, this method allows the generation of extremely uniform composite films, with nano-molecular scale design required for different applications such as sensors and catalysts [27,28].

The ISG method refers to a MOF film fabrication by immersing the substrate directly into the growth solution and the nucleation step and the growth step occur at the same time [29]. It has been widely used for fabrication of MOF based asymmetric membranes by depositing a MOF layer on both bare and surface modified porous substrates. However, an insufficient number of heterogeneous nucleation sites on the bare support and poor adhesion between the MOF layer and the substrate, may result in defective MOF films with inter-crystalline gaps between particles. Surface functionalization of substrates can effectively solve this issue by producing surface attached functional groups that act as anchors to ligands or metal ions for promoting heterogeneous nucleation and MOF film growth [30]. Hou et al. [23,31] reported zeolitic imidazolate framework-8 (ZIF-8) thin films on indium tin oxide (ITO) supports modified with 3-aminopropyltriethoxysilane (APTES) as a covalent linker between ZIF-8 crystals and ITO supports. Thus, this method provides a general route to fabricating ZIF membranes on porous supports. Until now, most of the reported MOF supported polymer membranes were focused on ZIF-8 and Copper benzene tricarboxylate (Cu_3_(BTC)_2_) (known as HKUST-1) thin films because these MOFs are both highly stable and easy to prepare at ambient temperatures using low boiling solvents like methanol, ethanol and water, and so limiting thermal or chemical damage to the polymer substrate materials [8,20]. The chemical structures of both ZIF-8 and HKUST-1 MOFs are given in Figure S1.

Growing ZIF-8 onto different polymer substrates has the potential to achieve high quality membranes for different applications. Jin et al. [18] deposited ZIF-8 on a polyimide membrane support for utilizing ZIF-8 as an efficient heterogeneous catalyst for the Knoevenagel reaction. Also, a thick continuous ZIF-8 membrane was crystallized on a highly porous flexible polysulfone surface using ISG synthesis [6]. Permeation tests of ZIF-8/polysulfone show high hydrogen separation performance, confirming the molecular sieving effect of ZIF-8. Recently, Shamsaei et al. [30] have prepared an ultrathin ZIF-8 membrane on the surface of bromomethylated poly(2,6-dimethyl-1,4-phenylene oxide) (BPPO) substrate after being modified with ethylene diamine (ED). The resulting ZIF-8/ED-modified BPPO membranes exhibited a significantly higher H_2_ permeance compared to other gases.

The growth of thin films of HKUST-1 on different functionalized polymer membranes has been of great interest for many applications. Thin films of HKUST-1 were deposited on the surface of the flexible organic polymer polyvinylacetate (PVAc), using the LbL growth method. The surface of PVAc was chemically modified to generate reactive carboxylic groups to enhance the compatibility between the HKUST-1 film and the polymer support, and to promote heterogeneous nucleation of crystal growth. An HKUST-1 film can also be grown on inert polymer surfaces, using a reactive layer acting as the nucleation centre [22]. Thus, HKUST-1 has been deposited on the inert polymers polyethylene (PE), polystyrene (PS) and polyvinylidene fluoride (PVDF), after being coated with a polydopamine layer. More recently, Li et al. [20] have prepared high quality layers of ZIF-8 and HKUST-1 on a PVDF membrane after PVDF surface modification by ammoniation (ammonia and ED), to produce a high density of amino groups that facilitate the heterogeneous nucleation for the MOF growth.

**Figure 1:**
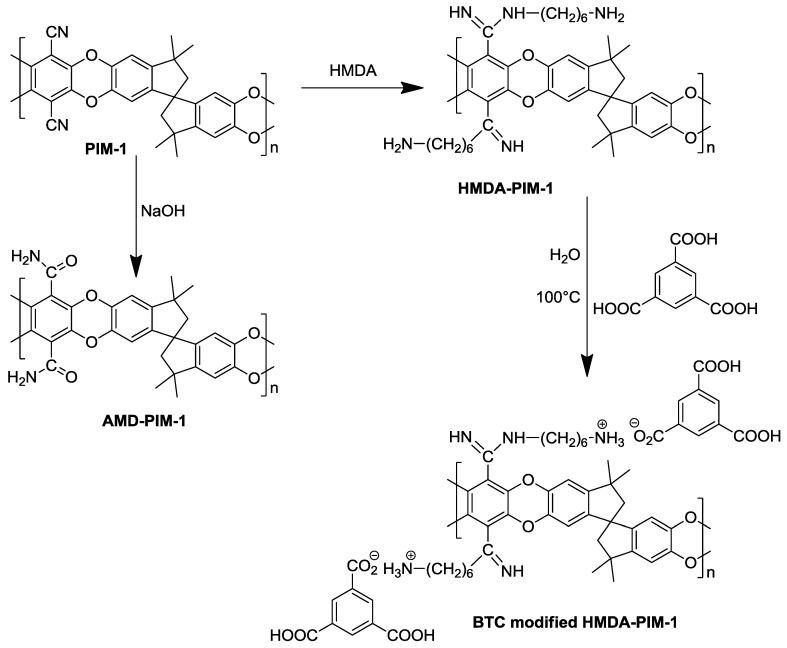
Functionalization of Polymer of Intrinsic Microporosity (PIM-1) to Amide (AMD)-PIM-1 by conversion of the nitrile group to an amide group. Possible products formed upon reaction of PIM-1 to HMDA-PIM-1 by conversion of the nitrile group to the imine, and further BTC modification of HMDA-PIM-1. An alternative reaction to the aminohexamethylamide (Ar-C(O)NHR, not shown) could take place simultaneously.

In this work, the PIM-1 membrane was chemically modified by converting the nitrile group to amide and to aminohexamethylamide (Figure 1), to favour adhesion of MOF particles on the membrane surface. The work further reports a series of experiments to grow ZIF-8 and HKUST-1 films onto the surface neat PIM-1 and modified PIM-1 membranes as a new approach to prepare gas separation membranes. The aim is to prepare sandwich-like asymmetric composite membranes based on PIM-1, which has itself already displayed good gas separation properties, with a MOF layer that adds additional selectivity to the system.

## 2. Results and Discussion

### 2.1. Membrane Preparation and Properties

#### 2.1.1. ZIF-8 on PIM-1 and Modified PIM-1 Membranes

**Figure 2:**
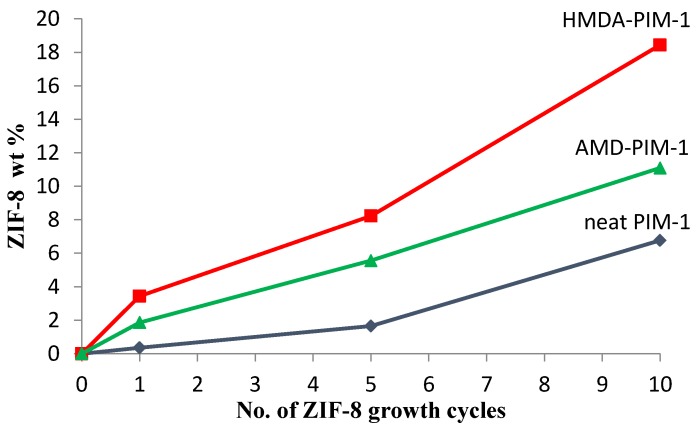
Amount of zeolitic imidazolate framework-8 (ZIF-8) deposited with increasing number of growth cycles on HMDA-PIM-1 (■), AMD-PIM-1 (▲) and neat PIM-1 membranes (◆).

The ZIF-8 particles were grown in situ onto the PIM-1 membranes under aqueous conditions at ambient temperature by addition of the precursors [Zn(NO_3_)_2_·6H_2_O and 2-methylimidazole] for ZIF-8 synthesis. The thickness of ZIF-8 layer on the surface polymer membranes can be tailored by repeated cyclic immersion of the previous ZIF-8/PIM-1 membranes into a fresh solution of the precursors of ZIF-8 synthesis for a number of times. In this study, ZIF-8 films were prepared on both neat PIM-1 and on hexamethylenediamine (HMDA)- and amide (AMD)-surface modified PIM-1 membranes to investigate the effect of the modification of the support. Figure 2 shows the amount of ZIF-8 deposited onto the three different supports as a function of the number of cycles, which increases in the order neat PIM-1 < AMD-modified PIM-1 < HMDA-modified PIM-1.

**Figure 4:**
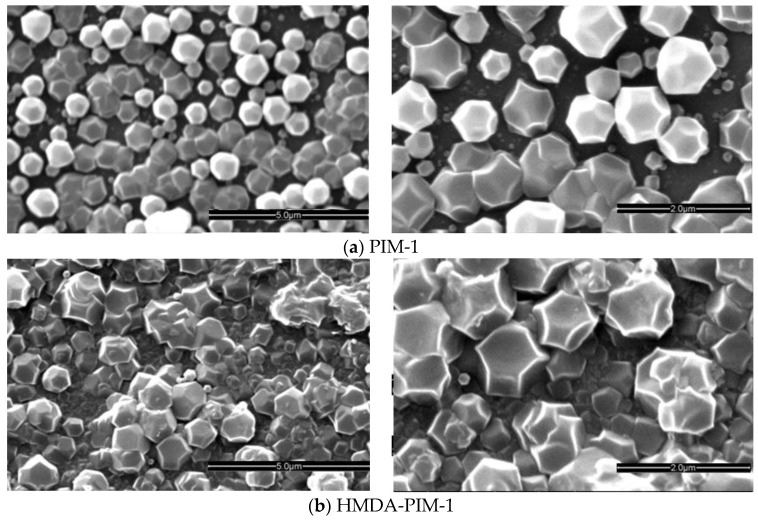

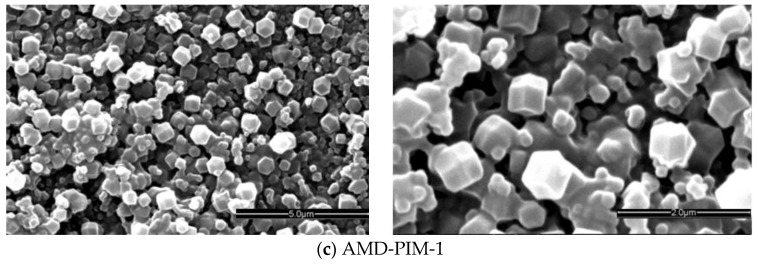
SEM images of ZIF-8 membranes supported on: (**a**) PIM-1 membrane; (**b**) HMDA-PIM-1 membrane and (**c**) AMD-PIM-1 membrane, all after five growth cycles.

Neat PIM-1 used as support for ZIF-8 particle growth must rely on the adhesion between the two materials due to the semi-organic nature of ZIF-8 and the polar cyano groups in the PIM-1 structure, and to further enhancement of the film integrity by inter-particle attraction [21]. However, interactions with the cyano groups are weak and it is difficult to control ZIF-8 crystallization on an unmodified PIM-1 film, thus leading to defects in the ZIF-8 supported polymeric membranes with many intercrystal voids (non-continuous film) at the beginning of ZIF-8 growth cycles (see Figure 4). As reported in the literature, repeating the growth cycle allows new ZIF-8 crystals to grow on previously existing ones, thus reducing inter-crystalline gaps (voids or defects) in the ZIF-8 film and increasing the ZIF-8 film thickness [21]. For all three supports, Figure 2 confirms a steady and almost linear increase in the amount of deposited ZIF-8 with the number of growth cycles. It also confirms the positive effect of the surface functionalization, which is reported in the literature to provide active sites for heterogeneous nucleation and to enhance the affinity between ZIF-8 film and the polymeric supports as well [26]. The weight percentage of ZIF-8 deposited on the surface of membranes based on HMDA-PIM-1 showed a stronger increase than that on AMD-modified PIM-1 because the terminal amino group of HMDA is more basic and interacts more strongly with Zn^2+^ and 2-methyl imidazole via hydrogen bonding or ionic interactions. In contrast, a much lower amount of ZIF-8 was deposited on the unmodified PIM-1 membrane after the first growth cycle, 0.35 wt %, compared to 1.86 wt % and 3.44 wt % for AMD-modified PIM-1 and HMDA-modified PIM-1 respectively.

**Figure 3:**
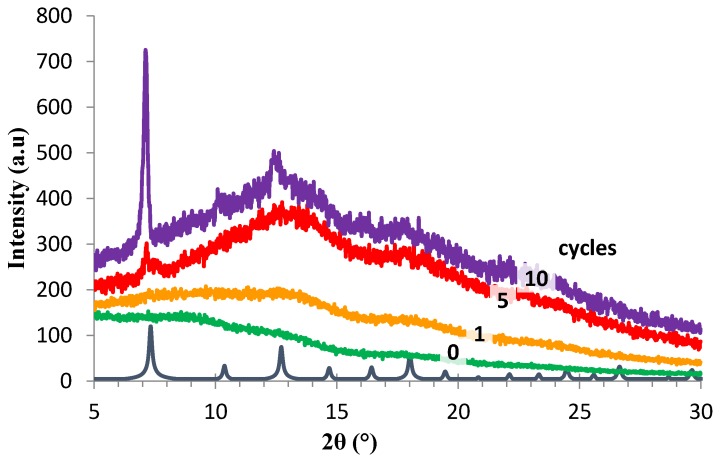
Wide angle powder XRD patterns of neat PIM-1 (green), and PIM-1-supported ZIF-8 membrane after 1 cycle (orange), 5 cycles (red) and 10 cycles (purple). Simulated ZIF-8 is shown for comparison (blue).

Figure 3 shows the powder X-ray diffraction (PXRD) pattern of the PIM-1 membrane after a number of ZIF-8 growth cycles. The XRD patterns match well with the simulated ZIF-8 pattern, confirming the presence of ZIF-8 on the samples. PXRD of the unmodified PIM-1 supported membrane showed it to be fully amorphous. The ZIF-8/PIM-1 composite membranes were analysed after 1, 5 and 10 cycles. After one cycle, the amount of ZIF-8 is too low to be detected using XRD [21]. The peak intensity increased as the number of growth cycles increased, in agreement with the observed increase in weight of the membrane (Figure 3). In the AMD-PIM-1 and HMDA-PIM-1 supports, a weak ZIF-8 signal already develops after the first cycle, and this becomes much stronger after the 5th and the 10th cycle (see Supplementary Figures S2 and S3) The chemical structures of the PIM-1 supported ZIF-8 membranes were further confirmed by ATR-IR spectroscopy of ZIF-8 powder, pristine HMDA-PIM-1 and ZIF-8/HMDA-PIM-1 composite membranes (Figure S4 in the SI). The HMDA-PIM-1 support shows a weak peak around 2220 cm^−1^, which implies that some unconverted nitrile groups are still present. This peak disappeared after the ZIF-8 thin film was deposited. The ZIF-8/HMDA-PIM-1 membranes show a weak bands around 3100 cm^−1^, assigned to the CH_3_ of the imidazole ring derived from 2-methyl imidazole.

The difference in growth of ZIF-8 crystals on the PIM-1, AMD-PIM-1 and HMDA-PIM-1 support membranes is clearly visible from the SEM images (Figure 4). On neat PIM-1, five cycles of synthesis were not enough to create a continuous film. Difficult control of ZIF-8 crystallization on unmodified PIM-1, due to the absence of suitable nucleation sites leads to few and relatively large crystals. This is reflected in defects in the ZIF-8 layer membranes with many intercrystal voids (gaps), as often observed in such cases [26]. The existing crystals may act to seed secondary growth on the previous particles, and eventually it may yield a continuous film after many cycles. On the contrary, crystal growth of the ZIF-8/HMDA-PIM-1 membrane produces crystal layers that are stacked on each other and this helped in covering completely the HMDA-modified PIM-1 support. Already after five cycles the ZIF-8 layer seemed like a continuous sheet and there are no visible macroscopic cracks or other defects. We cannot exclude with certainty that there are any defects sufficient for gas transport at the interfaces between crystals that are not visible by SEM images.

**Figure 5:**
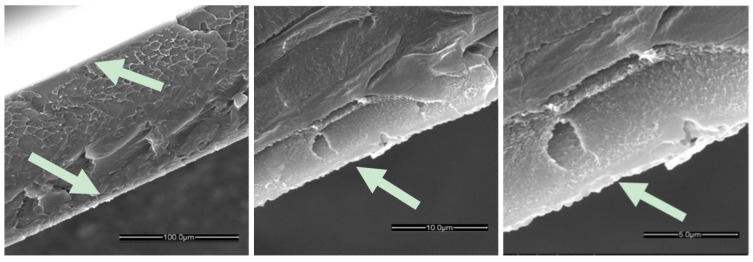
Cross section of the ZIF-8/HMDA-PIM-1 membrane after five ZIF-8 growth cycles. Arrow indicates the ZIF-8 layers.

**Figure 8:**
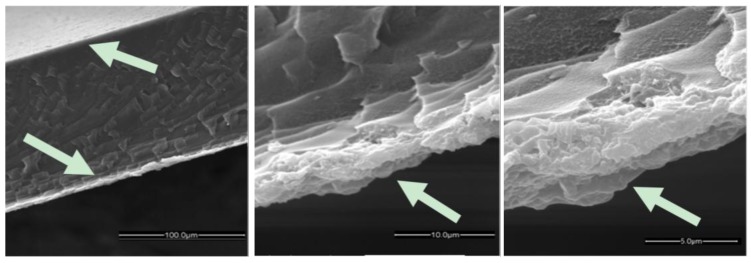
Cross-section of the HKUST-1/HMDA-PIM-1 membrane after five days of growth. The arrows indicate the HKUST-1 layer.

The ZIF-8 thin film adheres well to the HMDA-PIM-1 surface due to the attraction between the membrane and the ZIF-8 crystals and the inter-particle attraction, even after being washed several times with methanol. In contrast to the relatively poor heterogeneous nucleation of ZIF-8 crystals on neat PIM-1, the amino groups of the HMDA facilitate efficient nucleation of ZIF-8 [23]. An intermediate situation was achieved on the AMD-PIM-1 membrane. In this case, the AMD treatment clearly favours nucleation of ZIF-8 on the surface, but the film is not completely homogeneous and a nearly continuous polycrystalline ZIF-8 film is formed after five growth cycles, showing still numerous crystals of relatively small dimensions (Figure 4c). The cross section of the ZIF-8/HMDA-PIM-1 membrane reveals that the ZIF-8 thin films are composed of inter-grown crystals adhered tightly to the surface of the HMDA-PIM-1 support and after five cycles it has a thickness of about 5 μm (Figure 5). The crystals grow on both sides of the PIM-1 membranes, resulting in a sandwich-like composite membrane with no evident differences of thickness or morphology between the two sides of the membrane, as shown in the lower magnification SEM images in Figure 5 and Figure 8. No evident interface between the ZIF-8 layer and HMDA-PIM-1 support can be observed, confirming the good adhesion between the two phases. The chemical composition of the ZIF-8 thin films was further characterized by energy dispersive X-ray analysis (EDX) in the SEM instrument. The EDX spectrum of the ZIF-8 layer near the surface of the HMDA-PIM-1 membrane (Figure S5 in the SI) reveals the presence of a high Zn concentration, up to about 5 μm deep, in agreement with the ZIF-8 structure. Weaker signals of the elements C, N and O, originating from the polymer as well as the MOF, and Au from the sputter coating are visible as well.

#### 2.1.2. HKUST-1 Supported Surface Modified PIM-1 Membrane

**Figure 6:**
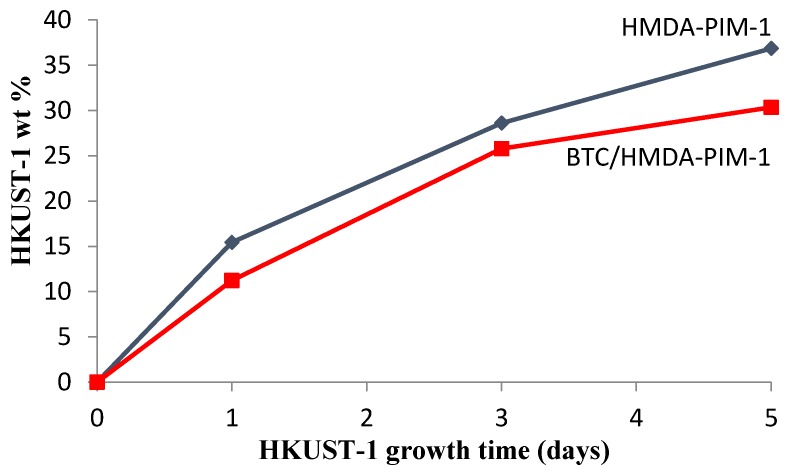
Amount of HKUST-1 deposited as a function of time on HMDA-PIM-1 (◆) and on BTC/HMDA-PIM-1 support membranes (■).

A first indication of the successful coating of the HMDA-PIM-1 and BTC/HMDA-PIM-1 supports with HKUST-1 after exposure to the growth solution [(Cu(OAc)_2_/benzene tricarboxylic acid (BTC)] was visible with the naked eye from the colour change from dark yellow to blue, whereas the bright yellow neat PIM-1 did not undergo a significant colour change. No significant growth of HKUST-1 on the unmodified PIM-1 membrane took place, even after five days. This must be attributed to the fact that the HKUST-1 cannot chemically bond to the PIM-1 support because there are apparently no suitable nucleation centres for HUKST-1 to grow on. Figure 6 shows the increase of the weight of HKUST-1 grown onto the surface of modified PIM-1 membranes as a function of time. The growth rate of HKUST-1 on the HMDA-PIM-1 support is only slightly higher than on the BTC/HMDA-PIM-1 support, and both show a gradual decrease in time.

**Figure 7:**
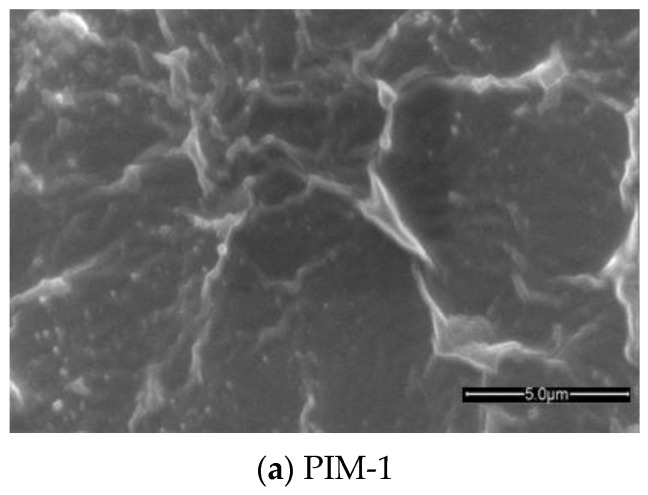

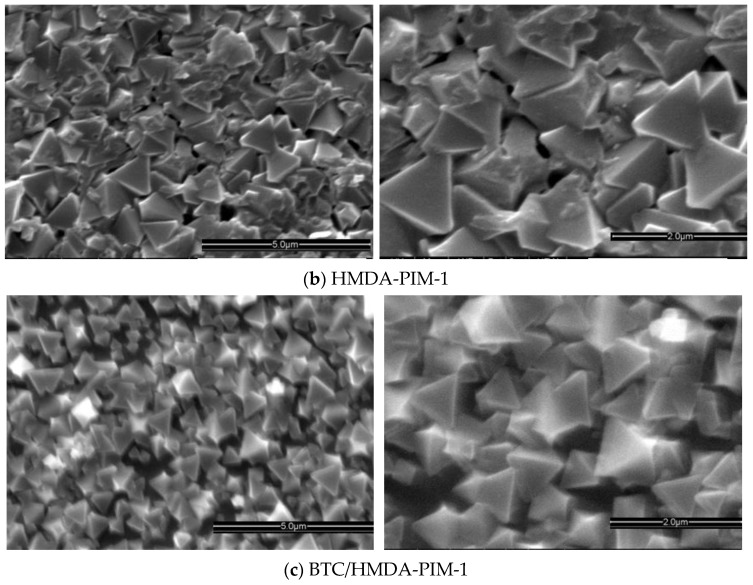
The XRD patterns of the HKUST-1 clearly reproduce the simulated pattern and confirm the formation of HKUST-1 (Figures S6 and S7 in the SI). The much sharper XRD patterns on the HMDA-PIM-1 than on the BTC/HMDA-PIM-1 membranes indicate the formation of larger and defect free crystals on the HMDA-PIM-1 supports. ATR-FTIR spectroscopy confirmed the presence of HKUST-1 on the surface of the HMDA-PIM-1 and BTC/HMDA-PIM-1 membranes, showing the typical signals of HKUST-1 at 700 cm^−1^, 1380 cm^−1^ and 1620 cm^−1^ in the same range for the sandwich membranes (Figure S8 in the SI).

Figure 7 shows the SEM images of the membrane surfaces, demonstrating the complete absence of HKUST-1 on the surface of the neat PIM-1 membrane and numerous densely packed crystals on the HMDA-PIM-1 and BTC/HMDA-PIM-1 supports. Whereas PIM-1 is inactive, the terminal amino group of HMDA on the surface of HMDA-PIM-1 membranes can react with BTC and/or coordinate with the copper ions of the HKUST-1 growth solution. In the case of the HKUST-1/modified PIM-1 membranes the HKUST-1 crystals are well inter-grown on the surface of these membranes and are intensely compacted together producing a dense layer (Figure 7b,c). This may be attributed to the strong attraction of the amino group of HMDA on the surface of HMDA-PIM-1 and the carboxylic group on the surface of BTC/HMDA-PIM-1 with the HKUST-1 precursor, acting as nucleation sites to grow a HKUST-1 thin film. The high density of heterogeneous nucleation sites is very important to fabricate a uniform and continuous film of HKUST-1 on the polymer surface.

The cross section of the HKUST-1/HMDA-PIM-1 membrane (Figure 8) shows that a film of HKUST-1 is intergrown firmly on the surface of the HMDA-PIM-1 support as a ca. 10 μm thick layer. As in the case of the ZIF-8 samples, the resulting membrane structure of HKUST-1/HMDA-PIM-1 forms a sandwich composite membrane, where the MOF is grown on both the sides of the support membrane. There is no clearly visible interface between the HKUST-1 layer and HMDA-PIM-1 support, confirming the good adhesion between the HMDA functionalized PIM-1 substrate and the HKUST-1 layer. A strong and sharp copper peak observed by SEM-EDX elemental analysis of the cross section of the HKUST-1 layer further confirms the presence of the HKUST-1 film deposited on the surface of modified PIM-1 membranes (Figure S9 in the SI).

### 2.2. Gas Transport Properties of MOF/PIM Sandwich Membranes

**Figure 9:**
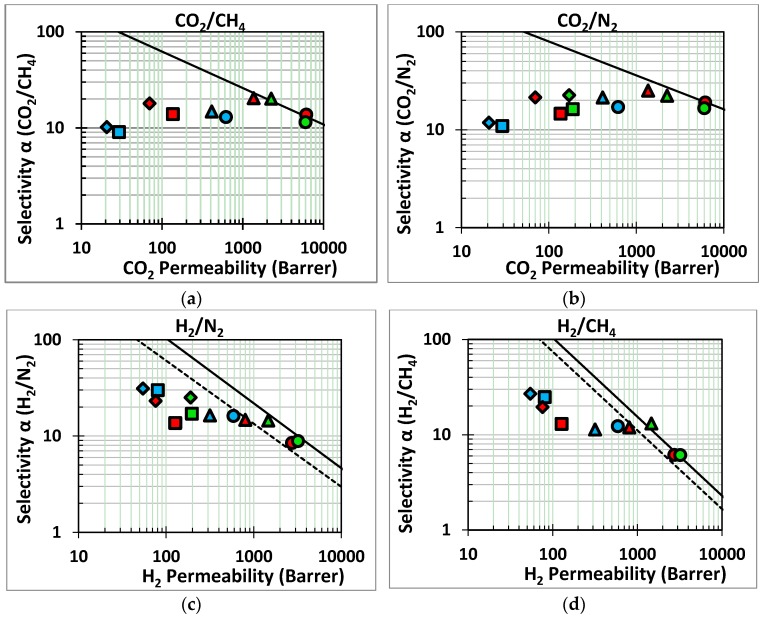
Robeson’s plots for four gas pairs of industrial interest. The dashed line and the solid line represents the Robeson 1991 upper bound [1] and the 2008 upper bound [2], respectively. Red series: neat PIM-1 (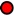) and ZIF-8/PIM-1 after 1(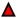), 5(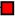) and 10(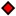) growth cycles; Green series: AMD-PIM1 (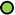) and ZIF-8/AMD-PIM-1 after 1(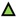), 5(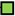) and 10(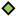) growth cycles; Blue series: HMDA-PIM1 (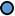) and ZIF-8/HMDA-PIM-1 after 1(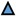), 5(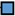) and 10(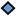) growth cycles.

The gas transport properties of the MOF/PIM sandwich membranes and their neat and modified supports were measured as the pure gas permeability and ideal selectivity, and the diffusion coefficient of each gas was obtained via time lag analysis. The results are summarized in the form of the well-known Robeson diagrams, [1,2] shown for some industrially relevant gas pairs in Figure 9. A complete overview of the individual data is reported in Tables S1–S5 of the supporting information. Surface modification of PIM-1 with HMDA caused a permeability reduction of approximately one order of magnitude with respect to neat PIM-1, whereas AMD-PIM-1 maintained nearly the same permeability. Subsequent ZIF-8 growth made all membranes more size selective, as can be seen in the Robeson diagrams from the increase in the H_2_/N_2_ selectivity and the H_2_/CH_4_ ideal selectivities. On the other hand, there is no significant change in the selectivity for gas pairs of similar size (e.g., CO_2_/CH_4_ or CO_2_/N_2_). This reduction in the permeability upon functionalization can be attributed to the grafting of large HMDA molecules on the PIM-1 and occupation of the free volume voids. On the other hand, the permeability remained nearly unaffected upon functionalization of PIM-1 with AMD.

Growth of ZIF-8 on the surface of these membranes leads to a decrease in the permeability of the membranes. In the case of PIM-1 and AMD-PIM-1 substrates after one growth cycle, the loss in permeability is balanced by an increase in selectivity. For instance, the ZIF-8(1Cycle)-AMD-PIM1 remains close to or exceeds the Robeson upper bound for the CO_2_/CH_4_ and the H_2_/CH_4_ gas pairs. Additional growth cycles do not further enhance the selectivity, while there is a continuous decrease in permeability. Unfortunately, the surface roughness of the ZIF-8/AMD-PIM1 membrane was so high after 5 and 10 cycles, that it caused a too high CH_4_ leak flow under the sealing ring in the membrane cell to allow reliable correction, and therefore the CH_4_ permeability and related selectivities could not be determined accurately for this sample.

**Figure 10:**
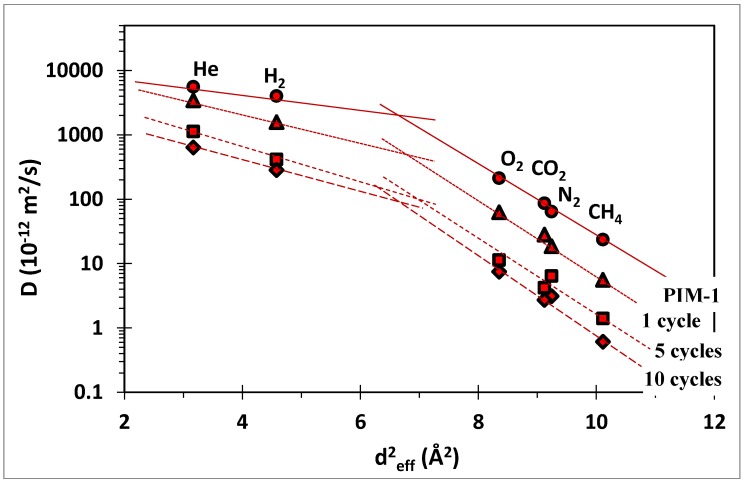
Effective diffusion coefficient versus the square effective diameter of the penetrant gases [32] for the neat PIM-1 and ZIF-8/PIM-1 membranes. Symbols refer to neat PIM-1 (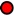) and ZIF-8/PIM-1 after 1(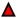), 5(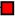) and 10(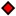) growth cycles.

The decrease in permeability is mainly due to a decrease in the diffusion coefficient of the gases through the membranes, as shown in Figure 10 for the neat PIM-1 based membrane as an example. The 10 growth cycles reduce the diffusion coefficient by almost 1 order of magnitude, in line with the decrease in permeability. The diffusion coefficient also decreases as a function of the molecular diameter of the gas, and the steepness of the correlation is a measure of the size selectivity of the membranes. It must be noted that the given diffusion coefficient is the effective diffusion coefficient, averaged over the three layers of the MOF-PIM-MOF sandwich structure. Therefore, the present analysis is valid only in qualitative terms, because a quantitative analysis would require application of a resistances in series model, and knowledge of the permeability of every single layer.

**Figure 11:**
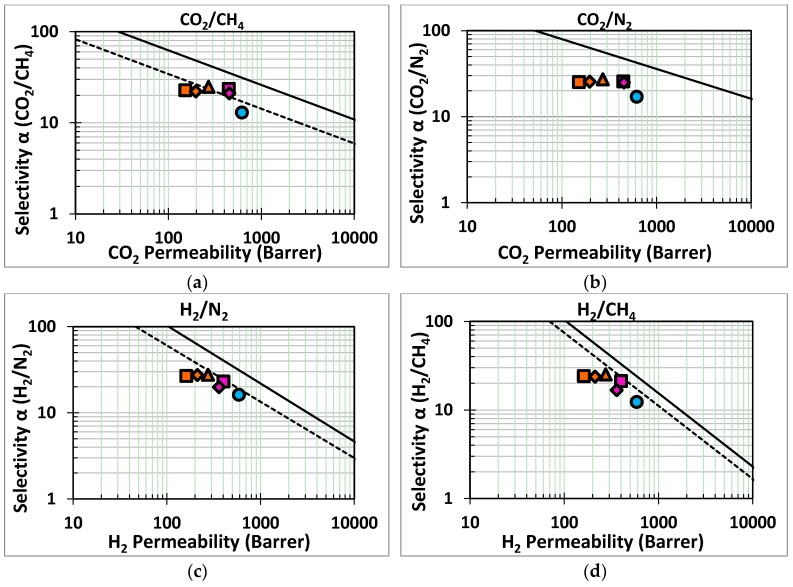
Robeson’s plots for four gas pairs of industrial interest. The dashed line and the solid line represents the Robeson 1991 upper bound [1] and the 2008 upper bound [2], respectively. Samples: HMDA-PIM1 (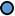); orange series: HKUST-1/BTC/HMDA-PIM1 after 1(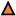), 3(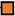) and 5(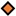) days of HKUST-1 growth; purple series: HKUST-1/HMDA-PIM1 after 3(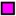) and 5(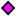) days of HKUST-1 growth.

The situation of the HKUST-1-based membranes is similar to that of the ZIF-8-based membranes. After an initial decrease of the permeability upon functionalization with HMDA and HMDA/BTC, the growth of HKUST-1 on HMDA-PIM1 or BTC/HMDA-PIM-1 leads to an increase in the selectivity as for all gas pairs reported Figure 11. However, contrary to ZIF-8, the increase in selectivity is not followed by a dramatic decrease in the permeability, especially in the case of the HMDA-PIM1 substrate. The slightly stronger decrease in the permeability for the membranes prepared using BTC/HMDA-PIM-1 substrates must be attributed to the presence of BTC groups on the membrane surface.

The membranes do not exceed the Robeson upper bounds for the indicated gas pairs. Nevertheless, the gain in selectivity without any loss in permeability for the HKUST-1/HMDA-PIM-1 membranes offers promising perspectives for the use of HKUST-1 on other polymers of intrinsic microporosity with functional groups capable to interact with the HKUST-1 without the pretreatment that reduces the permeability. The growth of the HKUST-1 on the membrane surface affects mostly the diffusion selectivity (α_(Dx/DN2)_ in Tables S4 and S5 in the SI). For instance, α_(DCO2/DN2)_ increases from about 0.6 for the HDMA-PIM-1 membrane to over 1.2 for the HKUST-1 covered membranes. This means that in the uncovered HDMA-PIM-1 the diffusion of N_2_ is faster than the CO_2_ diffusion, while the layer of HKUST-1 on the membrane surface overturns the situation, and the CO_2_ becomes the faster gas instead of N_2_.

## 3. Materials and Methods

All starting materials and solvents were purchased from Sigma-Aldrich, except for 5,5′,6,6′-tetrahydroxy-3,3,3′,3′-tetramethyl-1,1′-spirobisindane (TTSBI, 98%, Alfa, Heysham, UK), tetrafluoro-terephthalo-nitrile (TFTPN, 97%, Apollo Scientific Ltd., Bredbury, UK), anhydrous potassium carbonate (K_2_CO_3_, Fisher Chemical, Loughborough, UK), sodium hydroxide (NaOH, Fisher Chemical). All chemicals were used as received, apart from the following chemicals: 5,5′,6,6′-tetrahydroxy-3,3,3′,3′-tetramethyl-1,1′-spirobisindane (TTSBI) was dissolved in methanol and re-precipitated from dichloromethane before use. Tetrafluoro-terephthalo–nitrile (TFTPN) was purified by sublimation under vacuum before use.

All gases used for the permeability experiments were supplied by Pirossigeno (Castrolibero, Italy) at a minimum purity of 99.9995%.

### 3.1. Polymer and Membrane Synthesis

#### 3.1.1. PIM-1 Synthesis

PIM-1 was prepared according to the method described previously [33,34]. To a dry 500 mL three-necked round bottom flask under dry nitrogen gas, equipped with a Dean-Stark trap, 5,5′,6,6′-tetrahydroxy-3,3,3′,3′-tetramethyl-1,1′-spirobisindane (TTSBI) (17 g, 50 mmol), tetrafluoro­terephthalonitrile (TFTPN) (10 g, 50 mmol), anhydrous potassium carbonate (20.7 g, 150 mmol), dimethylacetamide DMAc (100 mL), and toluene (50 mL) were added. The reaction was carried out at 160 °C for 40 min. The product was then poured into methanol. The crude polymer was dissolved in chloroform and re-precipitated from methanol. The product was refluxed for six hours in deionized water and then dried at 100 °C for two days, yield 18 g (78%).

#### 3.1.2. PIM-1 Membrane Preparation

PIM-1 (0.192 g) was dissolved in anhydrous chloroform (10 mL) and stirred overnight. The resulting solution was filtered through glass wool into a levelled flat-bottom glass petri dish of diameter 7 cm inside a desiccator. The solvent was then allowed to slowly evaporate in order to form a membrane. The membrane was left for 3–5 days to complete solvent evaporation.

#### 3.1.3. Amide Modification of a PIM-1 Membrane

The recipe used to prepare the AMD-PIM-1 samples in this work, is based on the procedure reported by Satilmis et al. [35]. To a 100 mL two-necked round bottom flask under nitrogen, a number of pieces of PIM-1 membrane (approximately 2.5 cm diameter, 100 μm thickness) were mixed with an aqueous solution of sodium hydroxide (NaOH) (50 mL, 10 wt %). The reaction mixture was refluxed for 1 h for the first sample and 5 h for the second. After cooling, the excess NaOH solution was decanted and the modified PIM-1 membranes were washed several cycles in water and treated with a solution of dilute HCl. The resulting Amide-PIM-1 (AMD-PIM-1) membranes were soaked in methanol for 1 h and then allowed to dry at ambient temperature. Finally, the membranes were dried in a vacuum oven for 24 h at 60 °C. Recently, a new method was reported which gives a more selective reaction to the amide [36], but it was demonstrated that also with the present method no carboxylation of PIM-1 takes place under the given reaction conditions [35].

#### 3.1.4. HMDA Modification of a PIM-1 Membrane

A number of pieces of PIM-1 membrane (approximately 2.5 cm diameter, 100 μm thickness) were treated with an aqueous solution of hexamethylenediamine (HMDA) (50 mL, 10 wt %) in a 100 mL two-necked round bottom flask under nitrogen. The reaction mixture was refluxed at 120 °C for 6 h. When cooled, the excess HMDA solution was decanted. The modified PIM-1 membranes were washed several cycles in hot water until the pH became neutral, to remove unreacted HMDA. For further washing in ethanol, the membranes were stirred overnight, and then allowed to dry at ambient temperature. Finally, the membranes were dried in a vacuum oven for 24 h at 60 °C.

#### 3.1.5. BTC/HMDA Modified PIM-1 Membrane Preparation from HMDA Modified PIM-1 Membrane

To a 100 mL two-necked round bottom flask under nitrogen, some pieces of HMDA-surface modified PIM-1 membrane were mixed with 1 g of benzene tricarboxylic acid (BTC) and 50 mL water. The reaction mixture was heated under reflux for 1 h. After cooling, the excess BTC solution was decanted and the modified HMDA-PIM-1 membrane was washed with ethanol overnight to remove unreacted BTC. Finally, the membranes were dried in a vacuum oven for 24 h at 70 °C.

#### 3.1.6. Preparation of Membranes of ZIF-8 on PIM-1 and Modified PIM-1

For each of the PIM-1 membranes and the AMD- and HMDA-modified PIM-1 membrane substrates, a circular piece, approximately 2.5 cm in diameter, 100 μm in thickness, was vertically immersed to avoid sedimentation, in a vial (30 mL) containing a mixture of two solutions, 2-methyl imidazole (1.23 g, 0.015 mol) in deionized water (9 mL) and Zn(OAc)_2_·2H_2_O (0.055 g, 0.25 mmol) in deionized water (1 mL). The ZIF-8 growth solution quickly became cloudy; the growth took place at ambient temperature. The thin film of ZIF-8 supported PIM-1 membrane substrate was removed after 1 h from the vial and washed three times with methanol (one cycle). To increase the thickness of ZIF-8 film, the growth cycle above was repeated for 5 or 10 cycles with freshly mixed solutions of 2-methyl imidazole and Zn(OAc)_2_·2H_2_O. Finally, the ZIF-8 supported PIM-1 membrane was dried in a vacuum oven for 24 h at 60 °C. The pure ZIF-8 powder was collected from the vial after every cycle, washed, dried and used for characterization.

#### 3.1.7. Preparation of Membranes of HKUST-1 on HMDA and BTC/HMDA Modified PIM-1

A mixture of Cu(NO_3_)_2_·3H_2_O (2.96 g, 12 mmol) in 30 mL water and benzene tricarboxylic acid (BTC) (1.69 g, 8 mmol) in 30 mL ethanol were mixed in a jar (125 mL) and placed in an oven at 80 °C for 3 days. The resulting solution was cooled in an ice/water bath for 15 min and it was then filtered through a 0.22 μm PES membrane filter.

Circular pieces of PIM-1, HMDA-PIM-1 and BTC/HMDA-PIM-1 membrane, (approximately 2.5 cm diameter, 100 μm thickness) were placed vertically to avoid sedimentation in three separate vials (30 mL) containing the filtered HKUST-1 growth solution. The HKUST-1 growth took place at ambient temperature, and the membrane substrates were removed after 1 day, 3 days and 5 days. The thin film of HKUST-1 supported HMDA-PIM-1 and BTC/HMDA-PIM-1 membrane was washed three times with ethanol and dried in a vacuum oven for 24 h at 70 °C. The pure HKUST-1 powder was collected from the vial after every cycle, washed, dried and used for characterization.

### 3.2. Characterisation Techniques

#### 3.2.1. Structural Analysis

Powder X-ray Diffraction (PXRD) data of the MOF supported PIM-1 membranes were collected on a Panalytical X’ Pert Pro diffractometer Model PW3040/60 (Panalytical, Almelo, The Netherlands) using CuKα (λ = 1.5406 Å) radiation at an operating voltage of 40 kV and 30 mA at room temperature. A small amount of dry crushed powder sample was put onto the sample holder and scanned over the angular range of 2°–30° (2θ).

Scanning Electron Microscopy (SEM) analysis of the samples to study their morphology was performed using a FEI Quanta 200 ESEM (FEI, Hillsboro, NC, USA). The samples were coated with platinum via sputtering, using an Emitech coater (Emitech, Ashford, UK). Cross sections of membrane samples were prepared by freeze fracturing after immersion in liquid nitrogen. The fractured samples were mounted on stubs using two-sided conductive carbon adhesive tape. Energy Dispersion X-ray microanalysis was performed on the cross-section of the samples to obtain the elemental content of the coated MOF layer.

FT-IR spectra were recorded on a Bio-Rad FTS 6000 spectrometer (Bio-Rad, Watford, UK) equipped with an ATR setup, and annexed to a Whatman FTIR purge gas generator (Whatman, Little Chalfont, UK). The spectra were recorded in the attenuated total reflection (ATR) mode, with a resolution of 0.25 cm^−1^, a sensitivity of 1, and data from the average of 16 scans in the range 4000–500 cm^−1^.

#### 3.2.2. Pure Gas Permeation

Pure gas permeation experiments were performed on a fixed volume/pressure increase instrument constructed by Elektro & Elektronik Service Reuter (Geesthacht, Germany). The feed gas pressure was set at 1 bar, the permeate pressure was measured up to 13.3 mbar, or less, depending on the permeability. The gases were always tested in the same order (He, H_2_, N_2_, O_2_, CH_4_, and CO_2_). Nevertheless, this order was found to be irrelevant because a second cycle shows negligible differences compared to the first run. Circular membranes with an active area of 2.14 cm^2^ were tested at 25 °C. Permeabilities are reported in Barrer [1 Barrer = 10^−1^ cm^3^(STP) cm·cm^−2^·s^−1^·cm·Hg^−1^], and the diffusion coefficient was calculated from the so-called permeation time lag, *Θ*. Then the gas solubility coefficient is calculated in its approximate term as the ratio of the permeability over the diffusion coefficient. A more detailed discussion of the method can be found elsewhere [14].

## 4. Conclusions

Two series of PIM-1 based sandwich membranes were prepared successfully by coating ZIF-8 and HKUST-1 layers onto the surface of neat and functionalized PIM-1 membranes. The ZIF-8 layer was deposited on three membranes based on neat PIM-1, hexamethylenediamine-modified PIM-1 and amide-modified PIM-1. Different characterization techniques confirmed growth of a thicker and more continuous layer on HMDA-modified PIM-1. Neat PIM-1 yielded an incomplete coating of ZIF-8 and complete absence of HKUST-1 on its surface, apparently because the latter needs suitable reactive nucleation sites, while ZIF-8 can nucleate on the more inert cyano groups of neat PIM-1. The growth of ZIF-8 on the neat and modified PIM-1 membranes leads to a decrease of the permeability, and a modest increase in size selectivity. Additional growth cycles enhance the size selectivity at the expense of the permeability. For this reason, ZIF-8 is a less suitable MOF for this kind of application. Instead, HKUST-1 leads to a significant improvement of the selectivity, with a modest decrease in permeability. This work suggests that the use of PIMs with incorporated functional groups, which do not need functionalization before MOF growth on their surface may lead to more effective membranes.

## Supplementary Materials

The following are available online at http://www.mdpi.com/2077-0375/7/1/7/s1, Figure S1: Schematic representation of the ZIF-8 chemical structure (**a**) and HKUST-1 chemical structure (**b**); Figure S2: Wide angle powder XRD patterns of HMDA-PIM-1 (green), and HMDA-PIM-1-supported ZIF-8 membrane after 1 cycle (orange), 5 cycles (red) and 10 cycles (purple). Simulated ZIF-8 is shown for comparison (blue); Figure S3: Wide angle powder XRD patterns of AMD-PIM-1 (green), and AMD-PIM-1-supported ZIF-8 membrane after 1 cycle (orange), 5 cycles (red) and 10 cycles (purple). Simulated ZIF-8 is shown for comparison (blue); Figure S4: ATR-IR spectra of ZIF-8/HMDA-PIM-1 membrane, pure ZIF-8 powder and the HMDA-PIM-1 support membrane; Figure S5: EDX spectrum of the cross-section of ZIF-8 deposited on the HMDA-PIM-1 membrane after 5 growth cycles. The Au peak originates from the sputtering for SEM analysis; Figure S6: Wide angle powder XRD patterns of HMDA-PIM-1 (green), and HMDA-PIM-1-supported HKUST-1 membrane after 1 day (orange), 3 days (red) and 5 days (purple) of HKUST growth. Simulated HKUST-1 is shown for comparison (blue); Figure S7: Wide angle powder XRD patterns of BTC/HMDA-PIM-1 (green), and BTC/HMDA-PIM-1-supported HKUST-1 membrane after 1 day (orange), 3 days (red) and 5 days (purple) of HKUST growth. Simulated HKUST-1 is shown for comparison (blue); Figure S8: ATR-IR spectra of HMDA-PIM-1 membrane, pure HKUST-1 powder and HKUST-1/HMDA-PIM-1 membrane. The characteristic bands of HKUST-1 are located at 700, 1380 and 1620 cm^−1^; Figure S9: EDX spectrum of the cross-section of the HKUST-1 layer grown on the HKUST-1/HMDA-PIM-1 membrane for 5 days. The Pt peak originates from the sputtering for SEM analysis; Table S1: Transport data of sample PIM-1 and ZIF-8/PIM-1 sandwich membranes as a function of the number of ZIF-8 growth cycles; Table S2: Transport data of sample AMD PIM-1 and ZIF-8/AMD PIM-1 sandwich membranes as a function of the number of ZIF-8 growth cycles; Table S3: Transport data of sample HMDA PIM-1 and ZIF-8/HMDA PIM-1 sandwich membranes as a function of the number of ZIF-8 growth cycles; Table S4: Transport data of sample HKUST-1 BTC/HMDA-PIM-1 sandwich membranes as a function of the HKUST-1 growth time; Table S5: Transport data of sample HKUST-1 HMDA-PIM1 sandwich membranes as a function of the HKUST-1 growth time.
