# Supplementary material for: Synthesis and Transport Properties of Novel MOF/PIM-1/MOF Sandwich Membranes for Gas Separation
Source: Membranes (Basel). 2017 Feb 11;7(1):7. doi: 10.3390/membranes7010007 (PMC5371968; doi:10.3390/membranes7010007)
Supplement: Supplementary file 1 [file membranes-07-00007-s001.pdf]

Supplementary Materials: Synthesis and Transport Properties of Novel MOF/PIM-1/MOF Sandwich Membranes for Gas Separation

Alessio Fuoco, Muhanned R. Khdhayyer, Martin P. Attfield, Elisa Esposito, Johannes C. Jansen and Peter M. Budd

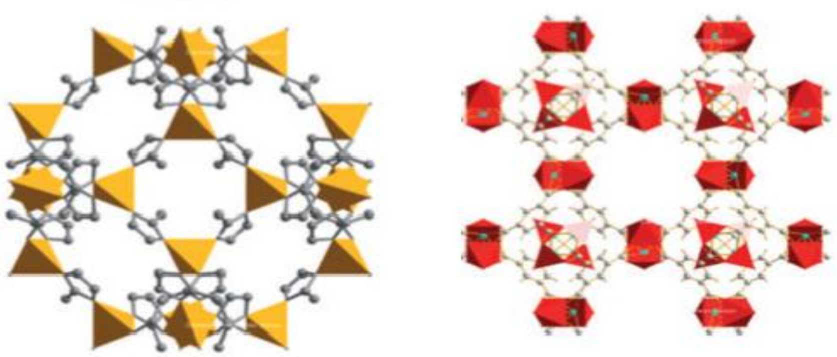

(**a**) (**b**)

Figure S1. Schematic representation of the ZIF-8 chemical structure (**a**) and HKUST-1 chemical structure (**b**).

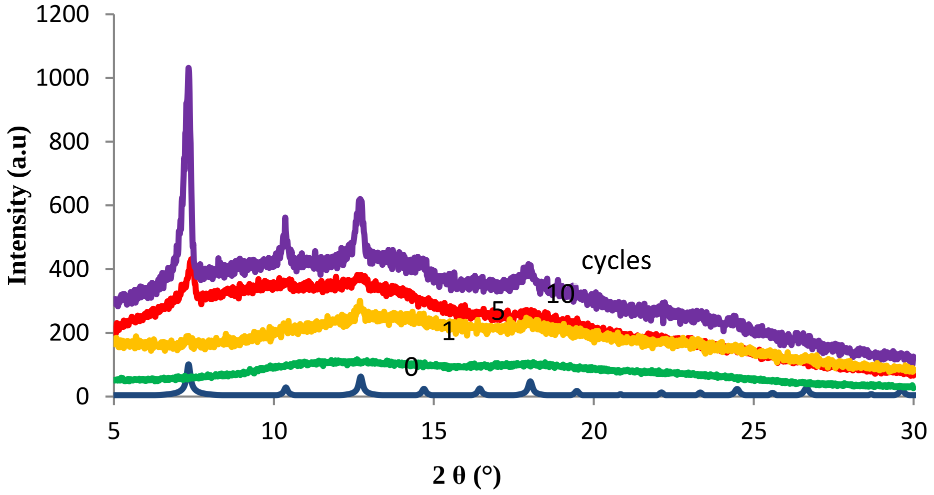

Figure S2. Wide angle powder XRD patterns of HMDA-PIM-1 (green), and HMDA-PIM-1-supported ZIF-8 membrane after 1 cycle (orange), 5 cycles (red) and 10 cycles (purple). Simulated ZIF-8 is shown for comparison (blue).

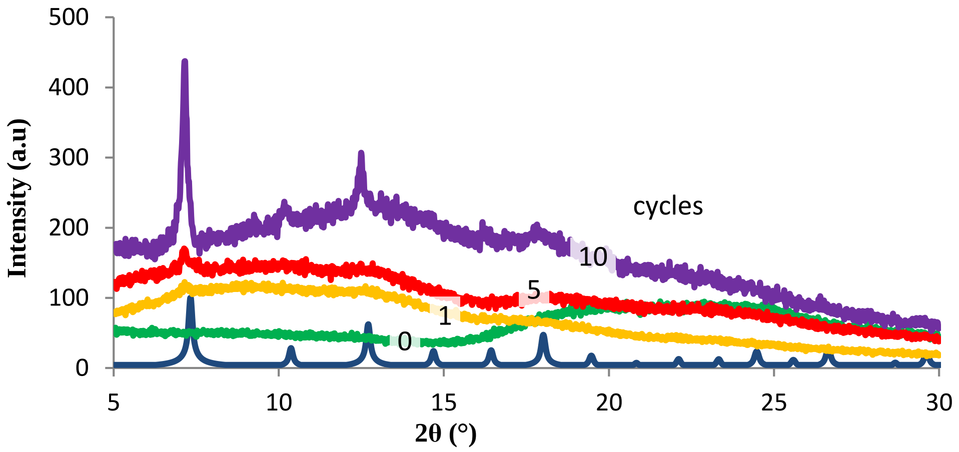

Figure S3. Wide angle powder XRD patterns of AMD-PIM-1 (green), and AMD-PIM-1-supported ZIF-8 membrane after 1 cycle (orange), 5 cycles (red) and 10 cycles (purple). Simulated ZIF-8 is shown for comparison (blue).

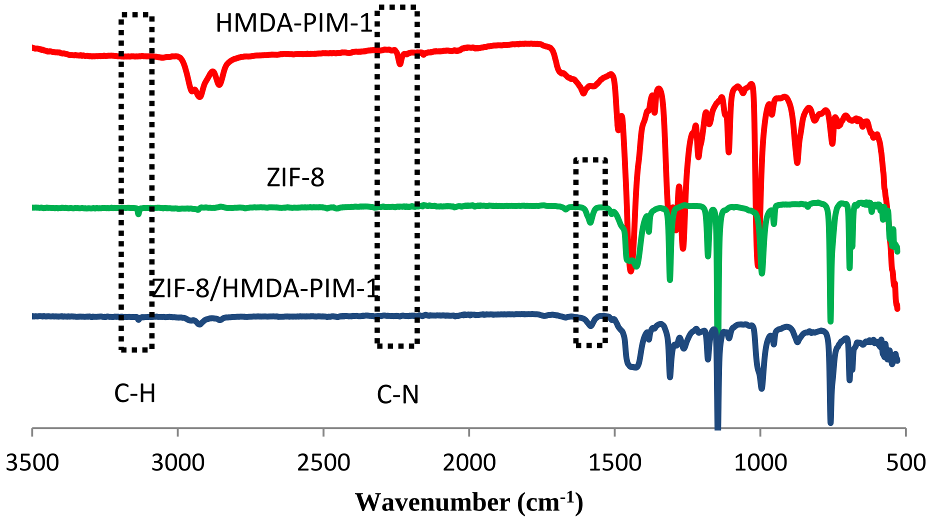

Figure S4. ATR-IR spectra of ZIF-8/HMDA-PIM-1 membrane, pure ZIF-8 powder and the HMDA-PIM-1 support membrane.

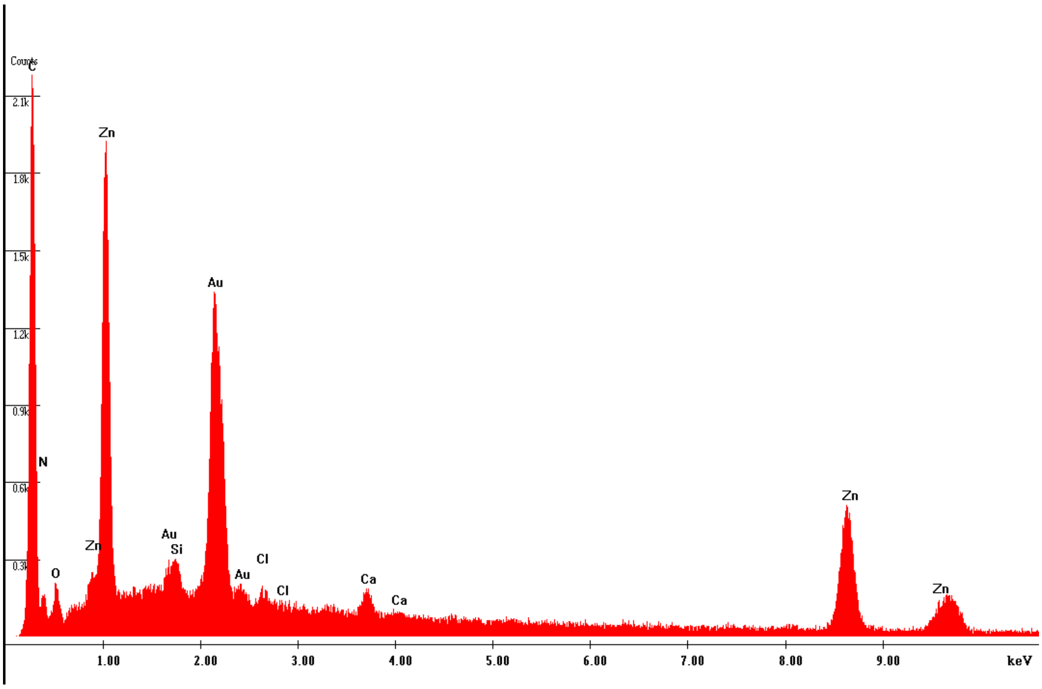

Figure S5. EDX spectrum of the cross-section of ZIF-8 deposited on the HMDA-PIM-1 membrane after 5 growth cycles. The Au peak originates from the sputtering for SEM analysis.

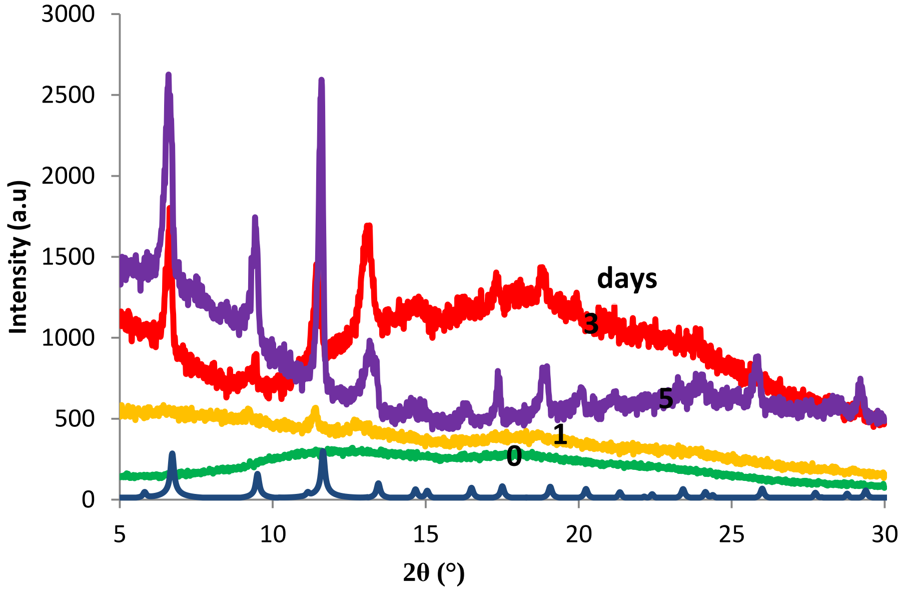

Figure S6. Wide angle powder XRD patterns of HMDA-PIM-1 (green), and HMDA-PIM-1-supported HKUST-1 membrane after 1 day (orange), 3 days (red) and 5 days (purple) of HKUST growth. Simulated HKUST-1 is shown for comparison (blue).

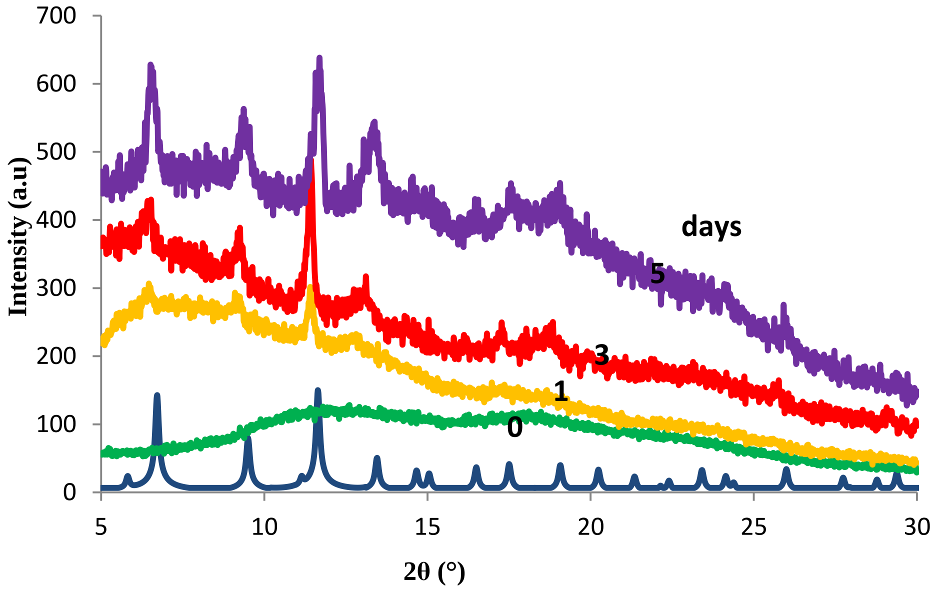

Figure S7. Wide angle powder XRD patterns of BTC/HMDA-PIM-1 (green), and BTC/HMDA-PIM-1-supported HKUST-1 membrane after 1 day (orange), 3 days (red) and 5 days (purple) of HKUST growth. Simulated HKUST-1 is shown for comparison (blue).

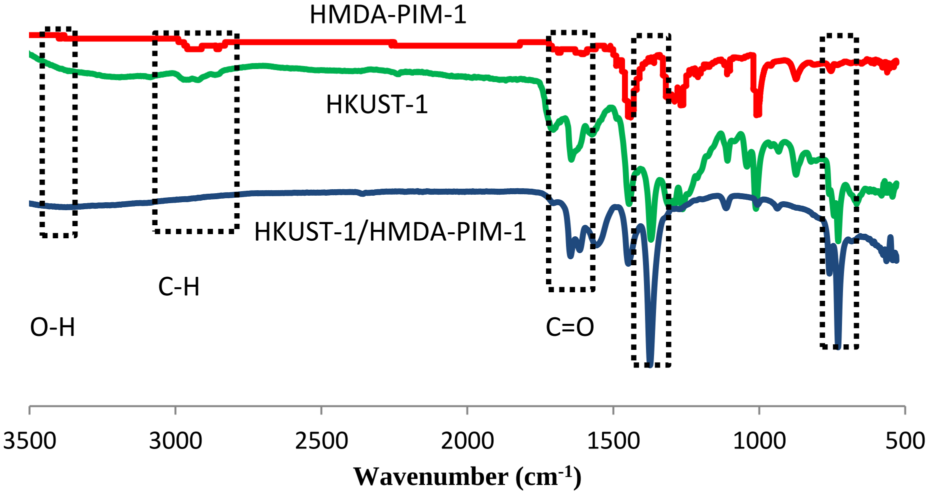

Figure S8. ATR-IR spectra of HMDA-PIM-1 membrane, pure HKUST-1 powder and HKUST-1/HMDA-PIM-1 membrane. The characteristic bands of HKUST-1 are located at 700, 1380 and 1620 cm^{-1}.

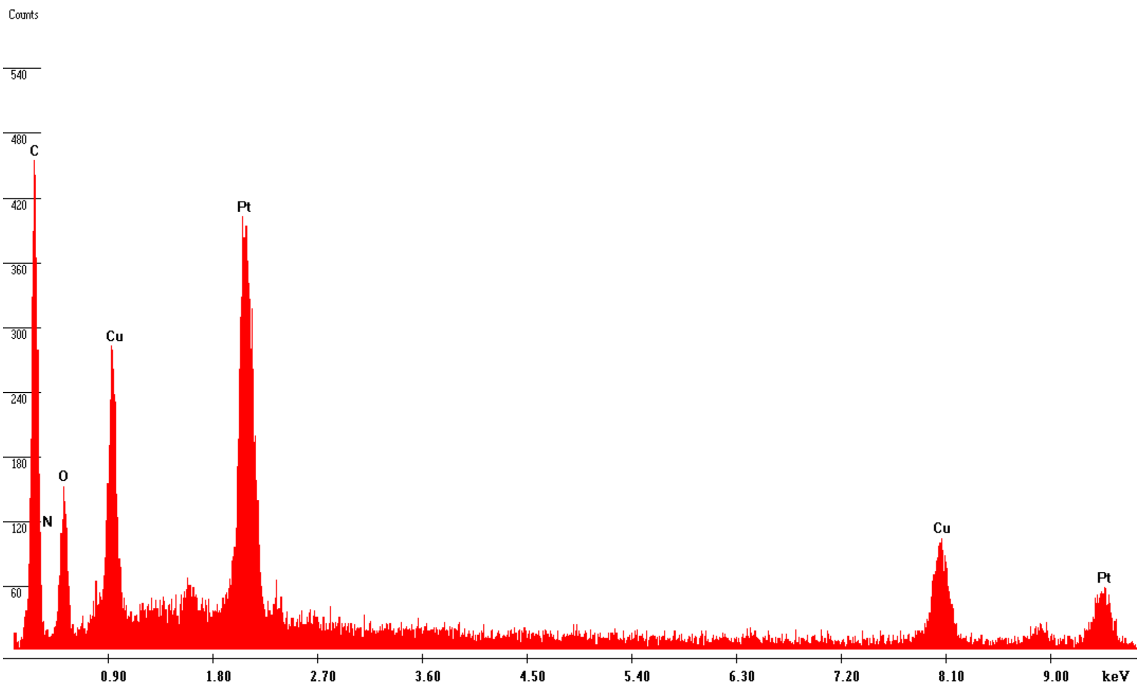

Figure S9. EDX spectrum of the cross-section of the HKUST-1 layer grown on the HKUST-1/HMDA-PIM-1 membrane for 5 days. The Pt peak originates from the sputtering for SEM analysis.

Table S1. Transport data of sample PIM-1 and ZIF-8/PIM-1 sandwich membranes as a function of the number of ZIF-8 growth cycles.

**Sample (cycles growth)**	**Gas Transport parameter**	**N_2**	**O_2**	**CO_2**	**CH_4**	**H_2**	**He**
Neat PIM-1	P_x [Barrer]	***323***	***1015***	***6132***	***447***	***2745***	***1169***
	α (P_x/P_{N_2})	-	3.14	18.94	1.38	8.48	3.61
	D_x [10^{-12} m^2·s^{-1}]	***64.0***	***212***	***86.2***	***23.5***	***4012***	***5587***
	α (D_x/D_{N_2})	-	3.32	1.35	0.37	62.7	87.4
	S_x [cm^3(STP) cm^{-3}·bar^{-1}]	***3.80***	***3.59***	***53.3***	***14.3***	***0.51***	***0.16***
	α (S_x/S_{N_2})	-	0.95	14.04	3.76	0.14	0.04
1 cycle	P_x [Barrer]	***54.5***	***215***	***1365***	***67.2***	***803***	***422***
	α (P_x/P_{N_2})	-	3.96	25.1	1.23	14.7	7.74
	D_x [10^{-12}·m^2·s^{-1}]	***18.6***	***62.5***	***28.2***	***5.59***	***1575***	***3452***
	α (D_x/D_{N_2})	-	3.36	1.51	0.30	84.7	185.5
	S_x [cm^3(STP)·cm^{-3}·bar^{-1}]	***2.20***	***2.59***	***36.31***	***9.02***	***0.38***	***0.09***
	α (S_x/S_{N_2})	-	1.18	16.54	4.11	0.17	0.04
5 cycles	P_x [Barrer]	***9.36***	***22.8***	***136***	***9.81***	***127***	***97.2***
	α (P_x/P_{N_2})	-	2.44	14.5	1.05	13.6	10.4
	D_x [10^{-12}·m^2·s^{-1}]	***6.37***	***11.3***	***4.20***	***1.40***	***414***	***1127***
	α (D_x/D_{N_2})	-	1.77	0.66	0.22	65.0	176.9
	S_x [cm^3(STP) cm^{-3}·bar^{-1}]	***1.10***	***1.51***	***24.28***	***5.27***	***0.23***	***0.06***
	α (S_x/S_{N_2})	-	1.37	22.04	4.78	0.21	0.06
10 cycles	P_x [Barrer]	***3.28***	***11.7***	***69.9***	***3.90***	***75.9***	***59.3***
	α (P_x/P_{N_2})	-	3.56	21.3	1.19	23.2	18.1
	D_x [10^{-12} m^2·s^{-1}]	***3.12***	***7.46***	***2.72***	***0.61***	***283***	***636***
	α (D_x/D_{N_2})	-	2.39	0.87	0.20	90.8	204
	S_x [cm^3(STP) cm^{-3}·bar^{-1}]	***0.79***	***1.17***	***19.25***	***4.79***	***0.20***	***0.07***
	α (S_x/S_{N_2})	-	1.49	24.4	6.07	0.26	0.09

Table S2. Transport data of sample AMD PIM-1 and ZIF-8/AMD PIM-1 sandwich membranes as a function of the number of ZIF-8 growth cycles.

**Sample (cycles growth)**	Gas Transport parameter	N_2	O_2	CO_2	CH_4	H_2	He
AMD PIM-1	P_x [Barrer]	***362***	***1107***	***6001***	***525***	***3215***	***1435***
	α (P_x/PN_2)	-	3.05	16.6	1.45	8.87	3.96
	D_x [10^{-12} $m^2 \cdot s^{-1}$]	***70.0***	***210***	***81.1***	***27.0***	***4230***	***6480***
	α (D_x/DN_2)	-	3.01	1.16	0.39	60.4	92.5
	S_x [cm^3(STP) $cm^{-3} \cdot bar^{-1}$]	***3.88***	***3.94***	***55.5***	***14.6***	***0.57***	***0.17***
	α (S_x/SN_2)	-	1.01	14.30	3.75	0.15	0.04
1 cycle	P_x [Barrer]	***101***	***405***	***2249***	***112***	***1467***	***716***
	α (P_x/PN_2)	-	4.00	22.2	1.10	14.5	7.07
	D_x [10^{-12} $m^2 \cdot s^{-1}$]	***20.8***	***77.1***	***28.5***	***6.20***	***1961***	***2562***
	α (D_x/DN_2)	-	3.71	1.37	0.30	94.2	123
	S_x [cm^3(STP) $cm^{-3} \cdot bar^{-1}$]	***3.65***	***3.94***	***59.1***	***13.5***	***0.56***	***0.21***
	α (S_x/SN_2)	-	1.08	16.2	3.71	0.15	0.06
5 cycles	P_x [Barrer]	***11.6***	***37.5***	***187***	*N/A* [a)]	***197***	***143***
	α (P_x/PN_2)	-	3.24	16.2	-	17.0	12.3
	D_x [10^{-12} $m^2 \cdot s^{-1}$]	***5.96***	***15.3***	***4.79***	*N/A* [a)]	***485***	***1475***
	α (D_x/DN_2)	-	2.56	0.80	-	81.5	247
	S_x [cm^3(STP) $cm^{-3} \cdot bar^{-1}$]	***1.46***	***1.84***	***29.3***	*N/A* [a)]	***0.30***	***0.07***
	α (S_x/SN_2)	-	1.26	20.1	-	0.21	0.05
10 cycles	P_x [Barrer]	***7.58***	***25.0***	***170***	*N/A* [a)]	***191***	***135***
	α (P_x/PN_2)	-	3.29	22.5	-	25.14	17.8
	D_x [10^{-12} $m^2 \cdot s^{-1}$]	***4.51***	***10.8***	***5.41***	*N/A* [a)]	***477***	***1024***
	α (D_x/DN_2)	-	2.40	1.20	-	106	227
	S_x [cm^3(STP) $cm^{-3} \cdot bar^{-1}$]	***1.26***	***1.73***	***23.6***	*N/A* [a)]	***0.30***	***0.10***
	α (S_x/SN_2)	-	1.37	18.7	-	0.24	0.08

[a)] The surface roughness of the ZIF-8/AMD-PIM1 membrane was so high after 5 and 10 cycles, that it caused a too high CH_4 leak flow of under the sealing ring in the membrane cell to allow reliable correction, and therefore the CH_4 permeability and related selectivities could not be determined accurately for these samples.

Table S3. Transport data of sample HMDA PIM-1 and ZIF-8/HMDA PIM-1 sandwich membranes as a function of the number of ZIF-8 growth cycles.

**Sample (cycles growth)**	**Gas Transport parameter**	N_2	O_2	CO_2	CH_4	H_2	He
HMDA PIM-1	P_x [Barrer]	***36.4***	***145***	***620***	***47.9***	***589***	***321***
	α (P_x/PN_2)	-	3.98	17.02	1.32	16.18	8.81
	D_x [10^{-12} $m^2 \cdot s^{-1}$]	***14.8***	***41.9***	***9.70***	***3.90***	***1136***	***2245***
	α (D_x/DN_2)	-	2.84	0.66	0.26	76.96	152.08
	S_x [cm^3(STP) $cm^{-3} \cdot bar^{-1}$]	***1.85***	***2.59***	***47.9***	***9.23***	***0.39***	***0.11***
	α (S_x/SN_2)	-	1.40	25.9	4.99	0.21	0.06
1 cycle	P_x [Barrer]	***19.3***	***73.9***	***412***	***27.9***	***316***	***191***
	α (P_x/PN_2)	-	3.82	21.3	1.44	16.3	9.85
	D_x [10^{-12} $m^2 \cdot s^{-1}$]	***10.5***	***31.4***	***9.02***	***3.48***	***838***	***1865***
	α (D_x/DN_2)	-	2.99	0.86	0.33	79.62	177
	S_x [cm^3(STP) $cm^{-3} \cdot bar^{-1}$]	***1.38***	***1.76***	***34.3***	***6.01***	***0.28***	***0.08***
	α (S_x/SN_2)	-	1.28	24.85	4.36	0.21	0.06
5 cycles	P_x [Barrer]	***2.69***	***11.3***	***29.3***	***3.25***	***80.6***	***64.0***
	α (P_x/PN_2)	-	4.19	10.9	1.21	29.9	23.8
	D_x [10^{-12} $m^2 \cdot s^{-1}$]	***2.08***	***5.57***	***0.71***	***0.50***	***234***	***692***
	α (D_x/DN_2)	-	2.68	0.34	0.24	113	333.4
	S_x [cm^3(STP) $cm^{-3} \cdot bar^{-1}$]	**0.97**	**1.52**	**31.1**	**4.83**	**0.26**	**0.07**
	α (S_x/SN_2)	-	1.56	32.0	4.97	0.27	0.07

10 cycles	P_x [Barrer]	***1.75***	***7.19***	***20.6***	***2.02***	***54.4***	***47.8***
	α (P_x/P_{N_2})	-	4.11	11.8	1.16	31.1	27.3
	D_x [10^{-12} m^2·s^{-1}]	***2.05***	***4.08***	***0.43***	***0.38***	***135***	***311***
	α (D_x/D_{N_2})	-	1.99	0.21	0.19	65.6	151
	S_x [cm^3(STP) cm^{-3}·bar^{-1}]	**0.64**	**1.32**	**35.5**	**3.95**	**0.30**	**0.12**
	α (S_x/S_{N_2})	-	2.07	55.5	6.19	0.47	0.18

Table S4. Transport data of sample HKUST-1 BTC/HMDA-PIM-1 sandwich membranes as a function of the HKUST-1 growth time.

**Sample (days growth)**	**Gas Transport parameter**	**N_2**	**O_2**	**CO_2**	**CH_4**	**H_2**	**He**
1 day	P_x [Barrer]	***9.97***	***46.9***	***271***	***10.9***	***275***	***182***
	α (P_x/P_{N_2})	-	4.71	27.2	1.10	27.6	18.2
	D_x [10^{-12} m^2·s^{-1}]	***2.61***	***10.1***	***3.62***	***0.67***	***380***	***893***
	α (D_x/D_{N_2})	-	3.87	1.39	0.26	145	342
	S_x [cm^3(STP) cm^{-3}·bar^{-1}]	***2.86***	***3.48***	***56.1***	***12.2***	***0.54***	***0.15***
	α (S_x/S_{N_2})	-	1.22	19.6	4.25	0.19	0.05
3 days	P_x [Barrer]	***6.04***	***26.4***	***152***	***6.70***	***161***	***116***
	α (P_x/P_{N_2})	-	4.37	25.2	1.11	26.7	19.3
	D_x [10^{-12} m^2·s^{-1}]	***2.18***	***7.43***	***2.76***	***0.49***	***325***	***692***
	α (D_x/D_{N_2})	-	3.41	1.27	0.22	149	318
	S_x [cm^3(STP) cm^{-3}·bar^{-1}]	***2.08***	***2.67***	***41.4***	***10.3***	***0.37***	***0.13***
	α (S_x/S_{N_2})	-	1.28	19.9	4.93	0.18	0.06
5 days	P_x [Barrer]	***7.73***	***34.9***	***197***	***8.93***	***212***	***147***
	α (P_x/P_{N_2})	-	4.52	25.5	1.16	27.5	19.0
	D_x [10^{-12} m^2·s^{-1}]	***3.82***	***13.1***	***4.47***	***1.03***	***459***	***1296***
	α (D_x/D_{N_2})	-	3.43	1.17	0.27	120	339
	S_x [cm^3(STP) cm^{-3}·bar^{-1}]	***1.52***	***1.99***	***33.0***	***6.48***	***0.35***	***0.09***
	α (S_x/S_{N_2})	-	1.32	21.8	4.28	0.23	0.06

Table S5. Transport data of sample HKUST-1 HMDA-PIM1 sandwich membranes as a function of the HKUST-1 growth time.

**Sample (days growth)**	**Gas Transport parameter**	**N_2**	**O_2**	**CO_2**	**CH_4**	**H_2**	**He**
1 day	P_x [Barrer]	***N/A***	***N/A***	***N/A***	***N/A***	***N/A***	***N/A***
	α (P_x/P_{N_2})						
	D_x [10^{-12} m^2·s^{-1}]	***N/A***	***N/A***	***N/A***	***N/A***	***N/A***	***N/A***
	α (D_x/D_{N_2})						
	S_x [cm^3(STP) cm^{-3}·bar^{-1}]	***N/A***	***N/A***	***N/A***	***N/A***	***N/A***	***N/A***
	α (S_x/S_{N_2})						
3 days	P_x [Barrer]	***17.5***	***74.2***	***447***	***19.0***	***402***	***249***
	α (P_x/P_{N_2})	-	4.25	25.6	1.09	23.0	14.2
	D_x [10^{-12} m^2·s^{-1}]	***4.70***	***16.3***	***5.98***	***1.09***	***727***	***899***
	α (D_x/D_{N_2})	-	3.48	1.27	0.23	155	191
	S_x [cm^3(STP) cm^{-3}·bar^{-1}]	**2.79**	**3.41**	**56.1**	**13.1**	**0.41**	**0.21**
	α (S_x/S_{N_2})	-	1.22	20.1	4.69	0.15	0.07
5 days	P_x [Barrer]	***18.1***	***74.8***	***453***	***21.5***	***360***	***222***
	α (P_x/P_{N_2})	-	4.13	24.98	1.19	19.87	12.26
	D_x [10^{-12} m^2·s^{-1}]	***6.62***	***22.0***	***8.70***	***1.88***	***720***	***1454***
	α (D_x/D_{N_2})	-	3.32	1.31	0.28	109	220
	S_x [cm^3(STP) cm^{-3}·bar^{-1}]	**2.05**	**2.56**	**39.1**	**8.61**	**0.38**	**0.11**
	α (S_x/S_{N_2})	-	1.24	19.0	4.19	0.18	0.06
